# TRIM Expression and Its Association With Disease Activity in Systemic Lupus Erythematosus

**DOI:** 10.1002/kjm2.70255

**Published:** 2026-06-23

**Authors:** Ling‐Ying Lu, Ling‐Jung Yen, Hui‐Ling Hsia, Zi‐Xeng Weng, Chi‐Hsiang Chung, Paul Wei‐Che Hsu, Chih‐Wei Chiu, Wu‐Chien Chien, Tsung‐Hsien Chang

**Affiliations:** ^1^ Division of Allergy, Immunology, and Rheumatology, Department of Medicine, Kaohsiung Veterans General Hospital Kaohsiung Taiwan; ^2^ Department of Medical Research, Tri‐Service General Hospital National Defense Medical University Taipei Taiwan; ^3^ School of Public Health National Defense Medical University Taipei Taiwan; ^4^ Institute of Molecular and Genomic Medicine, National Health Research Institutes Miaoli County Taiwan; ^5^ Department and Graduate Institute of Microbiology and Immunology National Defense Medical University Taipei Taiwan

**Keywords:** lupus nephritis, systemic lupus erythematosus, TRIM family

## Abstract

Systemic lupus erythematosus (SLE) is a chronic autoimmune disease with diverse manifestations, including rash, arthritis, and nephritis. Although autoantibodies are a key feature of SLE, their levels often poorly reflect disease severity, suggesting the involvement of additional contributing factors. The tripartite motif‐containing protein (TRIM) family, which regulates immunity, may play a role in SLE. Although TRIM21 is a known autoantigen, the roles of other TRIM family members remain unclear. The present study examined *TRIM* expression in SLE and evaluated the potential of TRIM proteins as biomarkers. *TRIM* expression in peripheral blood mononuclear cells was measured using reverse transcription quantitative polymerase chain reaction (PCR) and compared between patients with SLE (*n* = 33) and healthy controls (*n* = 20). Associations of *TRIM* expression with disease activity (active/inactive), renal involvement (nephritis/nonnephritis), clinical manifestations, and laboratory parameters were analyzed. The expression levels of several TRIM genes, specifically *TRIM5*, *TRIM11*, *TRIM21*, and *TRIM72*, were higher in patients with inactive SLE, nonnephritic disease, or low disease activity than in patients with active disease, nephritic disease, or relapsed SLE and healthy controls. The expression levels of these genes were considerably and negatively associated with clinical symptoms but not laboratory parameters. Hydroxychloroquine users exhibited higher levels of *TRIM21* expression than did nonusers. Overall, specific TRIM proteins are inversely correlated with SLE disease activity and clinical phenotypes, which indicates their potential as biomarkers. Hydroxychloroquine may regulate *TRIM21* expression. Further investigation into underlying mechanisms and continued TRIM monitoring are necessary to develop new SLE treatments and better assess disease progression.

**Abbreviations**
Cr: creatinine
CYC: cyclophosphamide
DPL: daily protein loss
GFR: glomerular filtration rate
HC: healthy control
LDA: low disease activity
LN: lupus nephritis
MMF: mycophenolate mofetil
PBMC: peripheral blood mononuclear cell
SLE: systemic lupus erythematosus
SLEDAI: Systemic Lupus Erythematosus Disease Activity Index
TRIM family: tripartite motif‐containing protein family

## 1 Introduction

Systemic lupus erythematosus (SLE) is a chronic, complex, and severe autoimmune rheumatic disease that affects multiple organ systems, such as the joints, skin, kidneys, lungs, serous membranes, and nervous system [1]. The global incidence of SLE ranges from approximately 5.14 (1.4–15.13) per 100,000 person‐years [2]. A nationwide population‐based epidemiology study in Taiwan reported that the prevalence and incidence of SLE were 42.2 per 100,000 individuals and 6.8 per 100,000 person‐years, respectively, in 2007 [3]. SLE has no known cure and imposes a substantial burden on both affected individuals and health‐care systems [4]. Although the etiology of SLE remains unclear, genetic, hormonal, and environmental factors may contribute to immune dysfunction, leading to autoantibody production and immune complex deposition [5].

Various autoantibodies associated with SLE include antierythrocyte, antidouble‐stranded DNA, anti‐Ro, anti‐La, antiphospholipid, anti‐Smith, and antinuclear ribonucleoprotein antibodies, which damage different organs and tissues [6]. However, autoantibody levels do not necessarily correlate with disease severity or treatment efficacy [7, 8]. Similarly, we found that the levels of known autoantibodies are not directly associated with SLE severity or treatment efficacy (data not shown). These findings highlight the presence of previously unidentified disease‐associated antigens or antibody‐related factors in patients with SLE.

The pathogenic role of adaptive immunity in SLE has been thoroughly investigated, and several mediators involved in adaptive immune responses have been targeted for therapy [9]. Although the role of innate immune responses in SLE pathogenesis remains insufficiently characterized, studies have demonstrated that innate immune cells and interferons contribute to the initiation and maintenance of SLE. Furthermore, genetic variations in innate immune components, such as melanoma differentiation‐associated protein 5, interferon‐regulatory factors (IRFs), and signal transducer and activator of transcription 4, determine the risk and severity of SLE [10].

The human tripartite motif‐containing protein (TRIM) family contains more than 70 members that are classified into 11 subfamilies on the basis of the presence of RING, B‐box, coiled‐coil, and other domains. The RING domain of TRIM proteins typically exhibits E3 ligase ubiquitination or SUMOylation activities, which may affect signal transduction in innate immunity [11]. Among TRIM family members, TRIM21/Ro52/SS‐A1—a 52‐kDa protein—is an autoantigen recognized by antibodies from patients with SLE and Sjögren's syndrome. Anti‐TRIM21 antibodies have been used as diagnostic markers for decades [12]. The biological activity of TRIM21 is crucial in innate and adaptive immunity. TRIM21 ubiquitinates IRFs and regulates type I interferon and proinflammatory cytokine production [13]. Spontaneous tissue inflammation and systemic autoimmunity mediated by the interleukin (IL)‐23‐T helper 17 cell pathway were observed in *TRIM21*‐deficient mice. These findings suggest that TRIM21 is a key negative regulator of inflammation and systemic autoimmunity [14, 15]. A study demonstrated a correlation between *TRIM21* expression and SLE disease activity, with higher TRIM21 mRNA and protein levels detected in peripheral blood mononuclear cells (PBMCs) from patients with SLE than in those from healthy controls (HCs) [16]. However, *TRIM21* expression across disease states in SLE remains to be examined. Thus, whether *TRIM21* and other TRIM family members are associated with SLE disease activity should be determined.

Given the emerging role of *TRIM21* in SLE pathogenesis, the present study analyzed the expression profile of the TRIM family in PBMCs from patients with SLE to evaluate the potential of TRIM proteins as biomarkers in SLE. The findings may highlight potential therapeutic targets for SLE treatment and inform improved approaches for disease prediction, early diagnosis and prognosis.

## 2 Materials and Methods

### 2.1 Study Cohort and Ethics Statement

Patients who met the 1997 American College of Rheumatology classification criteria for SLE and underwent regular follow‐up were identified by physicians from the Division of Allergy, Immunology, and Rheumatology at Kaohsiung Veterans General Hospital, a medical center in southern Taiwan. HCs were also enrolled. Clinical data were systematically collected and analyzed to ensure a comprehensive assessment. The study protocol was approved by the institutional review board of the study hospital (IRB/REC approval no. 18‐CT10‐13/180822‐2). This study adhered to the ethical principles outlined in the Declaration of Helsinki. Written informed consent was obtained from all participants before enrollment.

### 2.2 Specimens

PBMCs and serum samples were collected from all participants. A portion of each blood sample was sent to the institutional biobank for serum or plasma processing and long‐term storage at −80°C before further analysis. Isolated PBMCs were preserved in RNAlater or TRIzol to maintain RNA integrity and stability before total RNA extraction.

### 2.3 RNA Extraction and Gene Expression Analysis

Total RNA was extracted from PBMCs by using 500 μL of TRIzol reagent (ThermoFisher Scientific, Waltham, MA, USA) and 100 μL of 1‐Bromo‐3‐chloropropane, followed by centrifugation at 12,000*g* for 15 min at 4°C to separate the aqueous phase. RNA was precipitated from the aqueous phase by adding 250 μL of isopropanol and then pelleted through centrifugation. The RNA pellet was washed with 75% ethanol, air‐dried, dissolved in 30 μL of nuclease‐free water, and stored at −80°C. For complementary DNA synthesis, 1 μg of RNA was combined with 50 mM random primers and Superscript III reverse transcriptase (ThermoFisher Scientific) in a 12‐μL reaction volume, following the manufacturer's instructions. Quantitative PCR was performed using complementary DNA (6 ng), SYBR Green PCR master mix, and primers specific for the TRIM gene array (3 μM; Table S1) on an ABI StepOnePlus PCR system (Applied Biosystems, Foster City, CA, USA). The real‐time PCR protocol included initial activation at 95°C for 5 min, amplification was carried out over 40 cycles, consisting of denaturation at 95°C for 15 s, followed by a combined annealing and extension step at 60°C for 30 s. Real‐time PCR data were analyzed using StepOnePlus software (Applied Biosystems). The levels of mRNA expression were quantified using the 2^−ΔΔCt^ method and normalized to the expression level of glyceraldehyde‐3‐phosphate dehydrogenase.

### 2.4 Statistical Analysis

Relative expression of TRIM genes was calculated as fold changes (SLE vs. HCs) in gene expression and represented on a logarithmic scale. Identified transcripts were clustered using unsupervised hierarchical clustering on the basis of Pearson correlation coefficients. Both row and column clustering were performed using Euclidean distance and the complete linkage method (MultiExperiment Viewer, MeV v4.3) [17]. Differentially expressed genes were visualized. Statistical analyses were conducted using SPSS software (version 22.0; SPSS Inc., Chicago, IL, USA). Patient characteristics were examined and compared among different SLE groups by using baseline and outcome data. Categorical variables are presented as frequencies and percentages, whereas continuous variables are reported as mean ± standard deviation values.

Normality was assessed using the Kolmogorov–Smirnov test, with *p* < 0.001 indicating a nonnormal distribution. The Kruskal–Wallis test was used to determine differences in the relative expression of select TRIM genes among the study groups (HCs, symptomatic patients with SLE, and asymptomatic patients with SLE). Spearman correlation analysis was performed to identify the correlations between the relative expression of select TRIM genes and the clinical manifestations of SLE. The Mann–Whitney *U* test was used to compare *TRIM* expression between the groups on the basis of continuous laboratory parameters. Pearson correlation analysis was performed to determine the linear relationships between continuous laboratory parameters and *TRIM* expression. A *p* value of < 0.05 was considered statistically significant.

## 3 Results

### 3.1 Demographic Characteristics of Patients With SLE

The enrolled patients with SLE were divided into the following categories: fresh group, which comprised patients with a new diagnosis of SLE before treatment initiation; relapsed group, which comprised patients who had a major relapse after > 2 years of clinical remission and before aggressive therapy; refractory group, which comprised patients with chronic active disease and frequent flares who exhibited an inadequate response to standard‐of‐care treatment for major manifestations; and low disease activity (LDA) group, which comprised patients who remained in clinical remission or had LDA for > 3 years [18]. Disease activity over the preceding 4 weeks was examined using the SLE Disease Activity Index (SLEDAI). Patients with SLEDAI score > 4 and lupus‐related clinical features were classified as having active disease, whereas those with SLEDAI score ≤ 4 and no lupus‐related clinical features were classified as having inactive disease [19].

A total of 33 patients with SLE and 20 age‐matched HCs were recruited, and their mean ages were 31.1 ± 5.95 and 35.91 ± 11.02 years, respectively. Approximately two‐thirds of the patients were women. On the basis of SLEDAI scores, patients were classified into active and inactive disease groups. The mean SLEDAI scores in the active and inactive SLE groups were 15.08 ± 1.07 (*n* = 26; 78.79%) and 1.857 ± 0.55 (*n* = 7; 21.21%), respectively. Lupus nephritis (LN), a major complication of SLE, was observed in 22 (66.67%) of the 33 patients with SLE. Regarding clinical status, the SLE group included patients with new‐onset disease (*n* = 10; 30.30%), those with relapsed disease (*n* = 9; 27.27%), those with refractory disease (unresponsive to treatment; *n* = 7; 21.21%), and those with LDA (*n* = 7; 21.21%). The patients' clinicodemographic characteristics are summarized in Table 1.

**TABLE 1:** Clinicodemographic characteristics of patients with SLE and healthy controls.

Parameters	SLE patients (*n* = 33)	Healthy controls (*n* = 20)
Age (years)	31.1 ± 5.95	35.91 ± 11.02
Sex, *n* (%)		
Male	8 (24.24%)	7 (35.00%)
Female	25 (75.76%)	13 (65.00%)
Disease activity (SLEDAI), *n* (%)		
Active	26 (78.79%)	
Inactive	7 (21.21%)	
Active SLEDAI score (mean ± SD)	15.08 ± 1.07	
Inactive SLEDAI score (mean ± SD)	1.857 ± 0.55	
Lupus nephritis status, *n* (%)		
Nephritis	22 (66.67%)	
Nonnephritis	11 (33.23%)	
SLE diagnosis type, *n* (%)		
New‐onset	10 (30.30%)	
Relapse	9 (27.27%)	
Refractory	7 (21.21%)	
Low disease activity (LDA)	7 (21.21%)	

Abbreviation: SLEDAI, Systemic Lupus Erythematosus Disease Activity Index.

### 3.2 TRIM Expression in SLE

**FIGURE 1:**
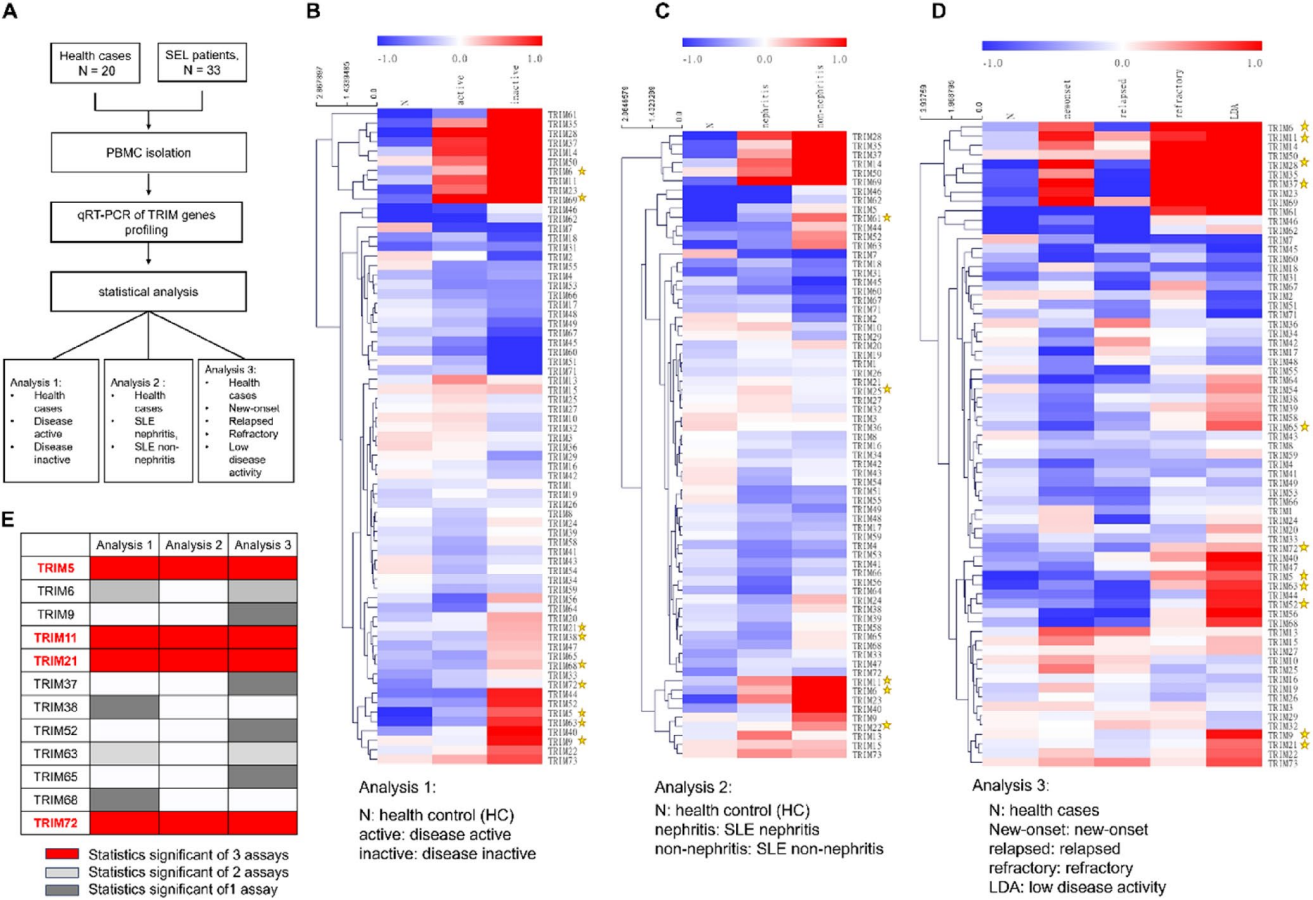
Differential *TRIM* expression between HCs and patients with various SLE states. (A) *TRIM* expression in PBMCs was analyzed using reverse transcription quantitative polymerase chain reaction and compared between HCs (*n* = 20) and patients with SLE (*n* = 33). Patients were stratified into three comparison groups: (1) HCs versus patients with active or inactive SLE (determined on the basis of SLEDAI scores); (2) HCs versus patients with LN SLE or non‐LN SLE; and (3) patients with various SLE states—new‐onset disease, relapsed disease, refractory disease, and LDA. (B) Heatmap analysis of *TRIM* expression in PBMCs from HCs (*n* = 20), patients with active SLE (*n* = 26), and patients with inactive SLE (*n* = 7; Analysis 1). (C) Heatmap analysis of *TRIM* expression in patients with SLE and nephritis (*n* = 22) and those with SLE but no nephritis (*n* = 11; Analysis 2). (D) Heatmap analysis of differential *TRIM* expression across the four SLE subgroups: Patients with new‐onset disease (*n* = 10), those with relapsed disease (*n* = 9), those with refractory disease (*n* = 7), and those with LDA (*n* = 7). (E) Box plots displaying the expression levels of *TRIM5*, *TRIM11*, *TRIM21*, and *TRIM72* in inactive, non‐LN, and LDA groups.

To determine whether *TRIM* expression is correlated with SLE disease activity, reverse transcription quantitative PCR‐based *TRIM* profiling was conducted using PBMCs isolated from the patients with SLE and HCs. The data were analyzed under three stratification conditions (disease activity, renal involvement, and clinical disease status) to examine the associations between *TRIM* expression and disease characteristics. In Analysis 1, *TRIM* expression was compared between HCs and patients with active or inactive SLE. In Analysis 2, patients with SLE were further classified into LN and non‐LN subgroups and compared with HCs. In Analysis 3, the SLE group was divided into four clinical subgroups on the basis of disease status: patients with new‐onset disease, those with relapsed disease, those with refractory disease, and those with LDA. These analyses clarified the potential associations between specific TRIM genes and different SLE disease phenotypes (Figure 1A).

### 3.3 TRIM Expression in Active and Inactive SLE

We first examined the correlations of *TRIM* expression with active and inactive disease states in patients with SLE. As shown in Figure 1B (Analysis 1, heatmaps), multiple TRIM genes were significantly upregulated in both the active and inactive SLE groups than in HCs. Several TRIM genes, such as *TRIM5*, *TRIM6*, *TRIM9*, *TRIM11*, *TRIM21*, *TRIM38*, *TRIM63*, *TRIM68*, and *TRIM72*, exhibited higher expression levels in the inactive SLE group than in both HC and active SLE groups. The upregulation of specific TRIM genes in the LDA group suggests their role in maintaining remission and regulating inflammation.

### 3.4 TRIM Expression in LN and Non‐LN SLE

LN is among the most severe manifestations of SLE and affects approximately 50% of all patients with SLE; it is associated with substantial morbidity and mortality [20]. To examine *TRIM* expression in LN, patients with SLE were divided into LN and non‐LN groups. *TRIM* expression in these groups was then analyzed and compared with that in HCs (Analysis 2). Heatmap analysis revealed that several TRIM genes, such as *TRIM6*, *TRIM9*, *TRIM11*, *TRIM14*, *TRIM23*, *TRIM35*, *TRIM37*, *TRIM40*, and *TRIM50*, exhibited higher expression levels in the non‐LN group than in the LN group. Therefore, these genes are associated with LN disease control. However, intergroup differences in only *TRIM9* and *TRIM11* reached statistical significance (Figure 1C). *TRIM5*, *TRIM11*, *TRIM21*, and *TRIM72* were significantly upregulated in patients with SLE and nephritis compared to HCs. However, these genes were similarly elevated in the non‐LN group, indicating that they do not serve as specific biomarkers for LN. Further investigation into the roles of TRIM family members in LN pathogenesis is warranted (Figure 1C).

### 3.5 TRIM Expression Across SLE Disease States

On the basis of clinical disease status, the SLE group was divided into four subgroups: patients with new‐onset disease, those with relapsed disease, those with refractory disease, and those with LDA. *TRIM* expression was compared between these groups and HCs (Analysis 3). Heatmap analysis revealed that T*RIM5*, *TRIM6*, *TRIM9*, *TRIM11*, *TRIM21*, *TRIM37*, *TRIM52*, *TRIM63*, *TRIM65*, and *TRIM72* were significantly upregulated in the new‐onset, refractory, and LDA groups. The expression levels of these genes were higher in the LDA group than in the other groups. Therefore, these TRIM genes contribute to disease control in SLE. By contrast, the upregulation of these genes was less pronounced in the relapsed group, suggesting that downregulation of the TRIM genes is associated with disease recrudescence (Figure 1D). Therefore, dysregulated *TRIM* expression is associated with loss of disease control and reactivation of SLE symptoms.

We further examined the results of Analyses 1–3 and determined that *TRIM5*, *TRIM11*, *TRIM21*, and *TRIM72* were consistently upregulated in the inactive, non‐LN, and LDA groups (Figure 1E). The Kolmogorov–Smirnov test revealed highly significant intergroup differences (*p* < 0.001) in the distributions of *TRIM5*, *TRIM11*, *TRIM21*, and *TRIM72*. Therefore, these four genes may modulate SLE disease activity, particularly during states of disease quiescence or low activity.

### 3.6 Correlation Between Clinical Data and TRIM Expression

We examined the correlations between the expression levels of *TRIM5*, *TRIM11*, *TRIM21*, and *TRIM72* and various clinical manifestations of SLE by using the Kruskal–Wallis test. Significant upregulation of *TRIM5*, *TRIM11*, *TRIM21*, and *TRIM72* was observed in most patients with SLE, regardless of the presence or absence of specific clinical manifestations (Tables 2 and 3 and Figure S1A).

**TABLE 2:** Relative expression of select TRIM genes in patients with SLE presenting with mucocutaneous manifestations.^a^

SLE clinical presentation (*n*, %)	*TRIM5*		*TRIM11*		*TRIM21*		*TRIM72*	
	Mean ± SD	*p* ^a^	Mean ± SD	*p* ^a^	Mean ± SD	*p* ^a^	Mean ± SD	*p* ^a^
**Acute skin: Malar rash**		**0.048**		**0.023**		0.421		**0.022**
Health control (20)	0.046 ± 0.050		0.0004 ± 0.007		0.141 ± 0.085		0.021 ± 0.009	
Malar rash (15, 45.45)	0.079 ± 0.046		0.002 ± 0.006		0.148 ± 0.068		0.040 ± 0.020	
Nonmalar rash (18, 54.54)	0.330 ± 0.640		0.005 ± 0.011		0.380 ± 0.900		0.091 ± 0.091	
**Chronic skin: *Discoid* lupus erythematosus (DLE)**		**0.014**		**0.015**		0.255		**0.011**
Health control (20)	0.046 ± 0.050		0.0004 ± 0.0004		0.141 ± 0.085		0.021 ± 0.009	
DLE (0)								
Non‐DLE (33, 100)	0.216 ± 0.484		0.004 ± 0.009		0.274 ± 0.668		0.068 ± 0.142	
**Photosensitive**		**0.048**		0.051		0.522		**0.037**
Health control (20)	0.046 ± 0.050		0.0004 ± 0.0004		0.141 ± 0.085		0.021 ± 0.009	
Photosensitive (8, 24.24)	0.076 ± 0.047		0.003 ± 0.008		0.149 ± 0.075		0.043 ± 0.025	
Nonphotosensitive (25, 75.76)	0.260 ± 0.551		0.004 ± 0.010		0.315 ± 0.765		0.076 ± 0.163	
**Hair loss**		**0.027**		**0.033**		0.476		**0.040**
Health control (20)	0.046 ± 0.050		0.0004 ± 0.0004		0.141 ± 0.085		0.021 ± 0.009	
Hair loss (7, 21.21)	0.356 ± 0.690		0.0009 ± 0.001		0.699 ± 1.441		0.143 ± 0.302	
Nonhair loss (26, 78.79)	0.178 ± 0.423		0.004 ± 0.010		0.160 ± 0.091		0.048 ± 0.046	
**Oral ulcer**		**0.028**		**0.043**		0.519		**0.038**
Health control (20)	0.046 ± 0.050		0.0004 ± 0.0004		0.141 ± 0.085		0.021 ± 0.009	
Oral ulcer (6, 18.18)	0.097 ± 0.037		0.004 ± 0.009		0.162 ± 0.060		0.032 ± 0.018	
Nonoral ulcer (27, 81.82)	0.242 ± 0.533		0.003 ± 0.009		0.299 ± 0.738		0.076 ± 0.157	

*Note:* The bold values indicate statistical significance (*p* < 0.05).

^a^ Kruskal–Wallis test.

**TABLE 3:** Relative expression of select TRIM genes in patients with SLE presenting with systemic and hematological manifestations.^a^

SLE clinical presentation (*n*, %)	*TRIM5*		*TRIM11*		*TRIM21*		*TRIM72*	
	Mean ± SD	*p* ^a^	Mean ± SD	*p* ^a^	Mean ± SD	*p* ^a^	Mean ± SD	*p* ^a^
**Fever**		**0.032**		0.053		**0.031**		**0.006**
Health control (20)	0.046 ± 0.050		0.0004 ± 0.0004		0.141 ± 0.085		0.021 ± 0.009	
Fever (11, 33.33)	0.251 ± 0.636		0.0006 ± 0.0004		0.116 ± 0.074		0.029 ± 0.024	
Nonfever (22, 66.67)	0.198 ± 0.405		0.005 ± 0.011		0.354 ± 0.811		0.087 ± 0.172	
**Arthritis**		**0.035**		**0.032**		0.462		**0.034**
Health control (20)	0.046 ± 0.050		0.0004 ± 0.007		0.141 ± 0.085		0.021 ± 0.009	
Arthritis (15, 45.45)	0.036 ± 0.692		0.004 ± 0.009		0.396 ± 0.989		0.091 ± 0.205	
Nonarthritis (18, 54.55)	0.097 ± 0.119		0.003 ± 0.010		0.174 ± 0.101		0.049 ± 0.052	
**Serositis**		**0.049**		0.050		0.515		**0.038**
Health control (20)	0.046 ± 0.050		0.0004 ± 0.0004		0.141 ± 0.085		0.021 ± 0.009	
Serositis (6, 18.18)	0.414 ± 0.857		0.006 ± 0.011		0.150 ± 0.060		0.042 ± 0.035	
Nonserositis (27, 81.82)	0.172 ± 0.369		0.003 ± 0.009		0.302 ± 0.737		0.074 ± 0.157	
**Neurological symptoms**		**0.039**		**0.049**		0.126		**0.033**
Health control (20)	0.046 ± 0.050		0.0004 ± 0.004		0.141 ± 0.085		0.021 ± 0.009	
Neurological (2, 6.06)	0.055 ± 0.054		0.0007 ± 0.0004		0.069 ± 0.074		0.030 ± 0.031	
Nonneurological (31, 93.94)	0.226 ± 0.498		0.004 ± 0.010		0.288 ± 0.687		0.070 ± 0.145	
**Leukopenia**		**0.005**		**0.039**		0.442		**0.019**
Health control (20)	0.046 ± 0.050		0.0004 ± 0.0004		0.141 ± 0.085		0.021 ± 0.009	
Leukopenia (22, 66.67)	0.173 ± 0.455		0.004 ± 0.010		0.151 ± 0.069		0.038 ± 0.030	
Nonleukopenia (11, 33.33)	0.301 ± 0.552		0.003 ± 0.008		0.522 ± 1.147		0.127 ± 0.239	
**Hemolytic anemia**		**0.049**		**0.028**		0.523		**0.007**
Health control (20)	0.046 ± 0.050		0.0004 ± 0.0004		0.141 ± 0.085		0.021 ± 0.009	
Hemolytic (19, 57.58)	0.301 ± 0.624		0.004 ± 0.011		0.360 ± 0.878		0.096 ± 0.184	
Nonhemolytic (14, 42.42)	0.100 ± 0.115		0.003 ± 0.006		0.158 ± 0.078		0.029 ± 0.022	
**Thrombocytopenia**		**0.010**		**0.015**		0.353		**0.038**
Health control (20)	0.046 ± 0.050		0.0004 ± 0.0004		0.141 ± 0.085		0.021 ± 0.009	
Thrombocytopenia (15, 45.45)	0.246 ± 0.555		0.006 ± 0.012		0.155 ± 0.105		0.057 ± 0.057	
Nonthrombocytopenia (18, 54.55)	0.191 ± 0.432		0.191 ± 0.432		0.374 ± 0.898		0.078 ± 0.188	

*Note:* The bold values indicate statistical significance (*p* < 0.05).

^a^ Kruskal–Wallis test.

Our results indicated negative correlations between the expression levels of select TRIM genes and mucocutaneous manifestations in patients with SLE. *TRIM5*, *TRIM11*, and *TRIM72* were downregulated in patients with malar rash and photosensitivity, whereas *TRIM5* and *TRIM72* were downregulated in patients with oral ulcers. *TRIM21* also exhibited a consistent trend, although nonsignificant, toward reduced expression in patients with the aforementioned manifestations. These findings suggest that reduced *TRIM* expression is associated with mucocutaneous involvement in SLE (Table 2 and Figure S1A).

The SLE group was further divided into patients with discoid lupus erythematosus and those without it. The discoid lupus erythematosus group exhibited significantly higher expression levels of *TRIM5*, *TRIM11*, and *TRIM72* than did HCs. Patients with hair loss exhibited higher expression levels of *TRIM5*, *TRIM21*, and *TRIM72* than did those without hair loss, with the differences in *TRIM5* and *TRIM72* reaching statistical significance. These findings suggest that TRIM gene expression varies with specific mucocutaneous manifestations in SLE (Table 2 and Figure S1A).

Compared with HCs, patients with SLE exhibited upregulation of *TRIM5*, *TRIM11*, *TRIM21*, and *TRIM71* across clinical manifestations such as fever, arthritis, serositis, neurological symptoms, leukopenia, hemolytic anemia, and thrombocytopenia. However, the expression of *TRIM21* did not significantly differ between patients with SLE and HCs (Table 3 and Figure S1B). Our analysis revealed an inverse relationship between *TRIM* expression and SLE disease activity. Higher expression levels of *TRIM5*, *TRIM11*, and *TRIM72* were significantly associated with lower incidence rates of fever, neurological symptoms, leukopenia, and thrombocytopenia. However, arthritis, serositis, and hemolytic anemia deviated from this pattern and were associated with elevated *TRIM* expression. These findings suggest that dysregulated *TRIM* expression is associated with systemic immune and hematological involvement in SLE, although substantial interindividual variability remains (Table 3 and Figure S1B).

Spearman rank correlation analysis was performed to identify the correlations between the expression levels of select TRIM genes (continuous variables) and categorical clinical features of SLE, such as the presence or absence of specific symptoms. The results revealed a significant, negative correlation between *TRIM21* expression and fever (Spearman *ρ* = −0.412, *p* = 0.017), indicating that patients presenting with fever tended to have reduced *TRIM21* expression. Similarly, a significant, negative correlation was observed between *TRIM5* expression and leukopenia/lymphopenia (*ρ* = −0.358, *p* = 0.041), suggesting that *TRIM5* is downregulated in patients with leukopenia, which may reflect altered immune regulation in these individuals (Table S2). These findings support the hypothesis that increased *TRIM* expression would be associated with less severe clinical phenotypes and would play a modulatory or protective role in SLE pathogenesis.

### 3.7 TRIM Expression in Relation to Clinical Variables in SLE

After the identification of altered expression levels of *TRIM5*, *TRIM11*, *TRIM21*, and *TRIM72*, we used the Mann–Whitney *U* test to compare the levels between patients stratified by continuous clinical laboratory parameters related to nephrotic syndrome. Renal function and proteinuria severity were assessed using 24‐h urinary total protein levels, calculated from the total daily urine volume. Additional laboratory parameters, such as complements (C3: 46.9 mg/dL; C4: 10.2 mg/dL), anti‐dsDNA antibodies (101 IU/mL), daily protein loss (DPL: 2.42 g/day), serum creatinine (0.98 mg/dL), and estimated glomerular filtration rate (GFR: 73.3 mL/min/1.73 m^2^), were also evaluated. Patients with SLE were divided into high and low groups by using the median value for each clinical parameter. The levels of *TRIM5*, *TRIM11*, *TRIM21*, and *TRIM72* expression were compared between the high‐ and low‐value groups.

A key finding of this study was the significant association between *TRIM72* expression and C4 level (*p* = 0.013). Patients with higher C4 levels exhibited significantly higher *TRIM72* expression (0.107 ± 0.199 vs. 0.031 ± 0.025 in those with lower C4 levels). This finding suggests an association between *TRIM72* expression and complement activity in SLE. *TRIM5* expression exhibited a trend toward significance in relation to C4 level (*p* = 0.056), with higher expression observed in patients with higher C4 levels (Table 4). Although this association was nonsignificant, the observed pattern warrants further investigation.

**TABLE 4:** Correlations of *TRIM* expression with various laboratory parameters in patients with SLE.^a^

Laboratory data (*n*)	*TRIM5*		*TRIM11*		*TRIM21*		*TRIM72*	
	Mean ± SD	*p* ^a^	Mean ± SD	*p* ^a^	Mean ± SD	*p* ^a^	Mean ± SD	*p* ^a^
**C3 (median = 46.9)**		0.407		0.589		0.150		0.746
Low (17, 51.52)	0.312 ± 0.660		0.0007 ± 0.0008		0.356 ± 0.931		0.081 ± 0.193	
High (16, 48.48)	0.113 ± 0.120		0.007 ± 0.013		0.188 ± 0.101		0.054 ± 0.055	
**C4 (median = 10.2)**		0.056		0.428		0.264		**0.013**
Low (17, 51.52)	0.083 ± 0.106		0.003 ± 0.007		0.143 ± 0.071		0.031 ± 0.025	
High (16, 48.48)	0.357 ± 0.669		0.005 ± 0.011		0.414 ± 0.952		0.107 ± 0.199	
**Anti‐dsDNA (median = 101)**		0.857		0.072		0.387		0.640
Low (17, 51.52)	0.207 ± 0.455		0.007 ± 0.012		0.397 ± 0.925		0.097 ± 0.196	
High (16, 48.48)	0.225 ± 0.529		0.0005 ± 0.0002		0.145 ± 0.063		0.037 ± 0.021	
**DPL (median = 2.42)**		0.589		0.449		0.471		0.130
Low (17, 51.52)	0.112 ± 0.150		0.005 ± 0.011		0.155 ± 0.105		0.045 ± 0.055	
High (16, 48.48)	0.320 ± 0.674		0.002 ± 0.007		0.401 ± 0.952		0.092 ± 0.197	
**Cr (median = 0.98)**		0.885		0.471		0.331		0.773
Low (17, 51.52)	0.201 ± 0.453		0.004 ± 0.008		0.360 ± 0.930		0.084 ± 0.193	
High (16, 48.48)	0.231 ± 0.530		0.003 ± 0.010		0.183 ± 0.104		0.501 ± 0.054	
**GFP (median = 73.3)**		0.829		0.183		0.494		0.719
Low (17, 51.52)	0.224 ± 0.514		0.002 ± 0.006		0.178 ± 0.104		0.044 ± 0.049	
High (16, 48.48)	0.207 ± 0.478		0.005 ± 0.012		0.376 ± 0.958		0.093 ± 0.199	

*Note:* The bold values indicate statical significance (*p* < 0.05).

Abbreviations: Cr, creatinine; DPL, daily protein loss; GFR, glomerular filtration rate.

^a^ Mann–Whitney *U* test.

The expression of other TRIM genes did not differ significantly between the high‐ and low‐value groups for C3, anti‐dsDNA, DPL, serum creatinine, and GFR. However, *TRIM11* expression tended to be lower in the high anti‐dsDNA group (*p* = 0.072), indicating an inverse correlation between *TRIM11* expression and autoantibody activity (Table 4). These findings suggest that specific TRIM genes, particularly *TRIM72*, are selectively associated with immunological or renal markers in SLE and warrant further investigation as potential mechanistic factors or biomarker candidates.

Pearson or Spearman correlation analyses indicated no significant correlations between the expression levels of *TRIM5*, *TRIM11*, *TRIM21*, and *TRIM72* and laboratory parameters associated with nephrotic syndrome, such as C3 and C4, DPL, serum creatinine, GFR, anti‐dsDNA, or anti‐SS‐A (anti‐Ro52/TRIM21; Table S3). These findings support the previously described negative association between *TRIM* expression and LN.

We further investigated the correlations between the use of specific medications and the expression levels of *TRIM5*, *TRIM11*, *TRIM21*, and *TRIM72* in patients with SLE. The following medications were analyzed: prednisolone, hydroxychloroquine (HCQ), and various immunosuppressants (e.g., mycophenolate mofetil, azathioprine, and cyclophosphamide). No significant correlation was observed between the use of prednisolone or immunosuppressants and the expression level of any of the four TRIM genes. However, HCQ use was significantly and positively correlated with *TRIM21* expression (Spearman *ρ* = 0.489, *p* = 0.004). No such correlation was noted between the use of HCQ and the expression of the other TRIM family members (*TRIM5*, *TRIM11*, or *TRIM72*; Table 5). These findings indicate that HCQ regulates *TRIM21* expression in SLE, highlighting a potential mechanism or biological consequence related to the known immunomodulatory effects of HCQ in the disease.

**TABLE 5:** Correlation between medication use and *TRIM* expression in patients with SLE.^a^

Treatment variables	*TRIM5*		*TRIM11*		*TRIM21*		*TRIM72*	
	Spearman's rho	*p*	Spearman's rho	*p*	Spearman's rho	*p*	Spearman's rho	*p*
Prednisolone	−0.055	0.759	−0.104	0.565	0.062	0.730	0.028	0.878
Hydroxychloroquine	0.116	0.189	−0.039	0.831	0.489	**0.004**	0.167	0.352
Immunosuppressants^b^	0.290	0.102	0.116	0.521	−0.013	0.943	0.258	0.148

*Note:* The bold values indicate statical significance (*p* < 0.05).

^a^ Spearman correlation.

^b^ Immunosuppressants included mycophenolate mofetil, azathioprine, and cyclophosphamide.

## 4 Discussion

This study provides valuable insights into the differential expression of the TRIM family in patients with SLE across different disease states and clinical manifestations. We determined that multiple TRIM genes were upregulated in the inactive SLE, non‐LN, and LDA groups and exhibited negative associations with specific clinical symptoms. We also observed that patients receiving HCQ treatment had significantly increased *TRIM21* expression. These findings implicate TRIM proteins in the regulation of immune responses and inflammation in SLE. Regular monitoring of *TRIM* expression in patients may provide valuable information regarding disease progression and treatment response. Further mechanistic investigation and continued *TRIM* expression monitoring are essential to support the development of novel SLE therapeutics and improve the assessment of disease progression.

Our cohort predominantly consisted of women, which aligns with the literature reporting a higher prevalence of SLE among women than among men [2]. Female hormones influence SLE risk, particularly during the reproductive years [21]. Various TRIM proteins positively or negatively regulate immune signaling [11] and have been implicated in autoimmune diseases [22]. Our stratified analyses revealed a correlation between *TRIM* expression and SLE disease status. We observed significantly higher *TRIM* expression in patients with inactive or non‐LN SLE than in patients with active SLE and HCs. The elevated expression of TRIM genes implicates them in the maintenance of immune homeostasis and prevention of disease flares, warranting further investigation into their functional roles in SLE pathogenesis.

LN affected approximately 66.67% of all patients in our cohort. Several biomarkers have been proposed for detecting and monitoring LN—for example, kidney‐specific molecules (FCGR3A, FCGR3B, and ACE), autoimmune pathology‐related molecules (CCR5, SPP1, and HLA‐DQA), serum proteins (C3 and C4), autoantibodies, and urinary protein markers (VCAM‐1, TNFR1, CXCR6, and MCP‐1) [23]. Additional LN‐related serum signatures include TNFR2, CD40, TIMP‐1, IL‐1β, IL‐1rA, SCF, and VEGF [24]. We identified another potential group of biomarkers. Several TRIM genes (*TRIM6*, *TRIM9*, *TRIM11*, *TRIM14*, *TRIM23*, *TRIM35*, *TRIM37*, *TRIM40*, and *TRIM50*) were upregulated in non‐LN cases. Their potential roles in LN disease control indicate that they can serve as useful biomarkers for LN monitoring. However, their roles in LN pathogenesis remain complex and require further investigation.

We observed significant upregulation of TRIM genes in the new‐onset and LDA SLE groups, with the LDA group exhibiting the highest expression. By contrast, the relapsed group exhibited less pronounced upregulation. These findings suggest that *TRIM* upregulation regulates disease activity and maintains symptom control in SLE, whereas reduced *TRIM* expression is correlated with disease recrudescence.

Given the proposed regulatory roles of *TRIM5*, *TRIM11*, *TRIM21*, and *TRIM72* in SLE, current evidence may support our findings. A genome‐wide gene expression analysis indicated that TRIM5 contributes to SLE pathogenesis through its effects on CD4+ T cells and macrophages [25]. *TRIM11* suppresses the differentiation of regulatory T cells, indicating its potential as a therapeutic target for modulating immune dysregulation associated with autoimmune diseases [26].

*TRIM21* dysregulation has been associated with SLE and Sjögren's syndrome. Increased TRIM21 transcript levels are often observed alongside elevated interferon responses. This observation appears paradoxical because *TRIM21* is expected to promote the ubiquitination of IRFs, thereby reducing interferon signaling [14]. A mouse study reported that *TRIM21* ubiquitinated and degraded STING, thereby downregulating type I interferon signaling and reducing induced or spontaneous lupus‐related pathology [27]. Thus, *TRIM21* may help maintain immune homeostasis by restraining excessive type I interferon signaling. This feedback mechanism may be crucial for preventing aberrant immune activation [28].

Because *TRIM21* is an autoantigen targeted by anti‐SSA/Ro antibodies, we investigated whether these autoantibodies affect its expression. Although our analysis revealed no direct correlation between anti‐SSA antibodies and *TRIM21* expression, a prior study suggested a complex interplay among type I interferon, TRIM21, and anti‐TRIM21 antibodies [16]. In HCs and anti‐TRIM21‐negative patients with SLE, type I interferon expression was inversely correlated with *TRIM21* level. By contrast, in anti‐TRIM21‐positive patients, interferon‐inducible genes were positively correlated with *TRIM21* expression. This regulatory feedback may be disrupted in the presence of anti‐TRIM21 antibodies; despite high *TRIM21* expression, type I interferon expression remains unsuppressed, suggesting that these antibodies neutralize or interfere with the physiological function of TRIM21 [16]. The autoantibodies may also destabilize TRIM21 or impair its function, thereby disrupting its role in the degradation of IRFs and STING. These findings highlight restoring the activity of TRIM21 and blocking the effects of anti‐TRIM21 antibodies as potential therapeutic strategies for SLE [13, 16, 27]. The findings further suggest that TRIM21‐mediated immune regulation is more complex than previously recognized; further investigation into its precise functional roles is warranted.

HCQ is an alkalinizing lysosomotropic agent that increases lysosomal pH, thereby inhibiting key cellular functions (e.g., autophagic influx) and suppressing type I interferon production. A study demonstrated the efficacy of HCQ in autoimmune diseases such as SLE [29]. In the present study, a significant correlation was observed between HCQ use and *TRIM21* expression in patients with SLE. TRIM21 is a type I interferon‐inducible protein. Therefore, the observed positive correlation between HCQ use and *TRIM21* expression appears contradictory. Direct clinical evidence of HCQ‐mediated regulation of *TRIM21* expression remains lacking. However, an in vitro study demonstrated that HCQ stimulates type I interferon and *TRIM21* expression, thereby promoting antiviral innate immune responses in J774A.1 macrophages [30]. Mechanistic investigations further revealed that HCQ blocks the degradation of autophagy‐related molecules, including p62 and LC3, thereby inducing the type I interferon production pathway [31]. These findings provide limited evidence consistent with our observation of a possible association between HCQ treatment and *TRIM21* expression. Further investigation of interferon production in our cohort may provide additional insight into this association. Although the precise mechanisms remain unclear, dysregulation of the autophagy pathway has been proposed as a contributing factor in autoimmune diseases, including SLE [32]. Several TRIM proteins, such as TRIM5, TRIM11, and TRIM21, regulate the autophagy pathway and contribute to cell damage and innate immunity [33]. Additional investigation into the mechanisms underlying SLE pathogenesis may clarify these relationships.

Although TRIM72 (also known as MG53) has been less extensively studied in autoimmunity, it has been associated with inflammatory regulation. TRIM72 suppresses inflammation by regulating NLRP3 and NF‐κB activity as well as intracellular calcium signaling [34, 35]. TRIM72 is an autoantigen, and patients with idiopathic inflammatory myopathies exhibit marked increases in anti‐TRIM72 levels [36]. Further investigation into the precise role of TRIM72 in SLE may guide disease management.

TRIM5, TRIM11, TRIM21, and TRIM72 share a conserved RBCC (RING‐B‐box‐coiled‐coil) architecture and, in most cases, a C‐terminal PRY/SPRY domain. The RING domain confers E3 ubiquitin ligase activity, which is essential for ubiquitination‐mediated proteasomal degradation and inflammation regulation. The B‐box and coiled‐coil domains are necessary for the formation of higher‐order protein structures that increase signaling activity [11]. The PRY/SPRY domain of TRIM21 binds intracellular antibody–antigen complexes, triggering rapid degradation and activating type I interferon production—a process associated with SLE [37]. Thus, the RBCC core plays a catalytic function across TRIM family members, whereas the variable C‐terminal domain modulates inflammatory responses, making the overall domain architecture critical for understanding pathogenic mechanisms in SLE.

A primary limitation of this study is its cross‐sectional design. Without serial samples collected across disease stages, we cannot definitively determine whether TRIM expression levels reflect changes in disease progression over time. Furthermore, the relatively small sample size (*n* = 33) and subsequent stratification into active and inactive SLE subgroups may have limited the statistical power of the study and affected the robustness of the findings. Nevertheless, the distinct expression patterns observed, along with their associations with clinical manifestations, highlight TRIM proteins as valuable biomarkers of disease activity. Further validation in larger, multicenter longitudinal cohorts is warranted.

This study focused on TRIM transcript levels in patients with SLE and HCs. Future studies should evaluate the activity of specific TRIM proteins in SLE. TRIM proteins play their regulatory functions primarily through the ubiquitination of other proteins, thereby affecting downstream pathways. Because TRIM proteins themselves have not been identified as risk factors in large‐scale genetic studies, the involvement of other critical factors or confounding variables not captured in this study cannot be excluded. Accordingly, our findings pertaining to *TRIM* expression are observational and should be interpreted with caution rather than being directly linked to SLE disease activity or phenotype. Another limitation of this study is the lack of data on crucial downstream signaling cascades, including the type I interferon pathway, inflammatory and antiinflammatory cytokine expression, and specific T‐cell populations (e.g., regulatory T cells). Despite the limitations, our study suggests key regulatory roles of the TRIM family in SLE pathogenesis. In the future, longitudinal studies should be conducted to assess the benefit of monitoring changes in *TRIM* expression for tracking disease progression in patients with SLE. In addition, future studies should investigate the mechanisms through which TRIM family members modulate immune responses, particularly the expansion of regulatory immune cell subsets, to elucidate the underlying antiinflammatory processes and their effects on disease progression. Ultimately, our findings may support the development of novel SLE therapeutics for preventing relapses and managing severe disease manifestations.

## Funding

This work was supported by Kaohsiung Veterans General Hospital (VTA108‐A‐3‐2).

## Conflicts of Interest

The authors declare no conflicts of interest.

## Supporting information


**Table S1:** Quantitative PCR array primers used for human TRIM genes.
**Table S2:** Correlation of the relative expression of select TRIM genes with SLE^a^.
**Table S3:** Correlations between *TRIM* expression and laboratory parameters in patients with SLE.^a^
**Figure S1:** Expression of TRIM genes across SLE clinical manifestations.

## Data Availability

The data that support the findings of this study are available from the corresponding author upon reasonable request.
