# Supplementary material for: TRIM Expression and Its Association With Disease Activity in Systemic Lupus Erythematosus
Source: Kaohsiung J Med Sci. 2026 Jun 23:e70255. Online ahead of print. doi: 10.1002/kjm2.70255 (PMC13399803; doi:10.1002/kjm2.70255)
Supplement: Supplementary file 1 — Table S1: Quantitative PCR array primers used for human TRIM genes. Table S2: Correlation of the relative expression of select TRIM genes with SLEa. Table S3: Correlations between TRIM expression and laboratory parameters in patients with SLE.a Figure S1: Expression of TRIM genes across SLE clinical manifestations. [file KJM2-9999-e70255-s001.docx]

**Supplementary Material**

***TRIM* Expression and Its Association With Disease Activity in Systemic Lupus Erythematosus**

**Supplementary Table S1. Quantitative PCR array primers used for human TRIM genes**

| Human TRIM gene accession number | QPCR primers |
| --- | --- |
| ***TRIM1, MID2***  *NM_012216* | F’- TTGCCGCCAGTGTCTTGA  R’- TTCAGGATATGCTCAGCTTGGTT |
| ***TRIM2***  *NM_001130067* | F’- TTAACACATCTGCTGACCCACTCTA  R’- TTCCAGAGTCTGCAACCACAAC |
| ***TRIM3***  *NM_006458* | F’- TGGACAAGCAGTTCCTGGTATG  R’- TGCAGGCAAGGAAGAACCTT |
| ***TRIM4***  *NM_033017* | F’- GCTCATCCCAAACTCGTCTTCT  R’- GAACTGGCTGATGCTGTATTTTTC |
| ***TRIM5***  *NM_033034* | F’- GCCTGGAACTCCTGACACAAC  R’- CATGGACTTCTTGTGGTTTGCA |
| ***TRIM6***  *NM_001003818* | F’- AAGGATGCTGCGAGTGTGTAGA  R’- GGTCACGTCAACCCAGTAGCTT |
| ***TRIM7***  *NM_203293* | F’- AAGCTCAGCAGCCAGATCCA  R’- TGCTCAGCGTGCTTTTGAATT |
| ***TRIM8***  *NM_030912* | F’- GAGCGAGAGCAGGACATTGAG  R’- ACTTCCTCCTTCAGTTGGTTCACT |
| ***TRIM9***  *NM_015163* | F’- TGGTCTTCACTTCAACAGCACATA  R’- CGGGCTGACTCCTGTTTTGT |
| ***TRIM10***  *NM_006778* | F’- AGCTTCCGGCCCAACTG  R’- TGGAGGCGCTCAATGTTCTC |
| ***TRIM11***  *NM_145214* | F’- GGATGCGTTGCTGTTCCAA  R’- CCACCATCTTCTGCCACAAGA |
| ***TRIM13***  *NM_005798* | F’- GGGAGCACACCAAACATGTCT  R’- GGCATCCCTTTCCTGAGCAT |
| ***TRIM14***  *NM_014788* | F’- CGTGGAAGCCGTGGAGAGTA  R’- AGCTGGCAGTTTATGCTTTCCT |
| ***TRIM15***  *NM_033229* | F’-GCCCGCTGGGAGAAACTT  R’-CAGAGGAACTCGGCATCGTT |
| ***TRIM16***  *NM_006470* | F’-CCTTCTGCTGCCCTGATCA  R’-TGCATCCAGGGAGACTATGGT |
| ***TRIM17***  *NM_016102* | F’- ACCAAGGTGGCCGAGATG  R’- TCCTGGCACAGGTCTTGCTT |
| ***TRIM18, MID1***  *NM_000381* | F’-TGTGACGAGTGCCTGAAAGC  R’-TCAGACGATGGCCTGTAAAGG |
| ***TRIM19, PML***  *NM_033238* | F’-GAAGAAGTACAGCCGCTATCTAAGC  R’-CCAGAGCCTGCAGGTTGAAA |
| ***TRIM20, MEFV***  *NM_000243* | F’- CGACCCGCCTGCTAATAAAG  R’- CTTCCAACTCTGTAGTCCACGAAGA |
| ***TRIM21, RO52***  *NM_003141* | F’- TGTGCCCAGTCTCGGAAAC  R’- TCCTGTGCAGCCTCCTCAA |
| ***TRIM22***  *NM_006074* | F’- CCACGGAGCACTCATCTACAAG  R’- CTAGGCAGTTCCAAGGATTGAAA |
| ***TRIM23, ARD1***  *NM_001656* | F’- GAACAAATCGTGGAAGATGGAAT  R’- CGAATACATGACCGGGCATT |
| ***TRIM24, TIF1A***  *NM_015905* | F’- ACCGCCTCCACGTTTGATAA  R’- GGTGGATATCTCAGTTGTTGAGGAT |
| ***TRIM25***  *NM_005082.4* | F’- GACCACGGCTTTGTCATCTTC  R’- AAAGTCCACCCTGAACTTATACATCA |
| ***TRIM26***  *NM_003449* | F’- ACCAACGCAGAGTCACAGGAA  R’- GCCACTTGAGCCACAGGAA |
| ***TRIM27***  *NM_006510* | F’- CGGCAAGTGCGGTACAGTTA  R’- GACACAGGGAAACAGATTGAACCT |
| ***TRIM28, KAP1, TIF1B***  *NM_005762* | F’- AAGGACCATACTGTGCGCTCTAC  R’- ACGTTGCAATAGACAGTACGTTCAC |
| ***TRIM29***  *NM_012101* | F’- AAGGACGACCTGCTCAATGTATG  R’- GGTCCGCCTTGCACATCT |
| ***TRIM31***  *NM_007028* | F’- TTGTGAACAAACTGCAAGAGGAA  R’- ACAGTCGATGGTGACAGGTTTCT |
| ***TRIM32***  *NM_012210* | F’- TGGCTCTGTAGGCCCTGATG  R’- AGCAATGCAGCGGAAATCC |
| ***TRIM33, TIF1G***  *NM_015906* | F’- CACCACCTCTCTCAACCAACCT  R’- TTCCAGGCTTGCCAATGC |
| ***TRIM34***  *NM_021616* | F’- GTCCTGTGTGTGGTATCAGTTACTCA  R’-CCACTATGTTGGCCAGATGCT |
| ***TRIM35***  *NM_171982* | F’- TGCTGGCACATGAGATCGA  R’- CGGCTCTTGTGTTTCATGAGAA |
| ***TRIM36***  *NM_018700* | F’- GAACTTGAAGAGAGACCGTGTAGAGA  R’- GGATGCGTTCTGCAGCAA |
| ***TRIM37***  *NM_015294* | F’- GCGGGTTTCACGACTCCTT  R’- TTCACCATCAGGAAAACTGGAA |
| ***TRIM38***  *NM_006355* | F’-TCAACCACCAGCACCAAGAA  R’-ATGCTTACTGGGTTCGTCATCA |
| ***TRIM39***  *NM_021253* | F’- ACCGCAGTCTCCGACCTAATC  R’- CCTGGAGCTGCTTGGCAAT |
| ***TRIM40***  *NM_138700* | F’- AGCCTGGTCATTGATCTGGAAA  R’- CACCAGCATTCTTCAGTGTGTTG |
| ***TRIM41***  *NM_033549* | F’- CCAACGGCAAACGCTATCA  R’- AAAGCGCCTTGGTTTCTCACT |
| ***TRIM42***  *NM_152616* | F’- CCTCGGCAACCAGCACAT  R’- TTCTTGTCAGTGTTGCTGAAGGA |
| ***TRIM43***  *M_138800* | F’- ACTCACCTGCGTCATCTGTTTG  R’- AGGCAGAGACAGGGCCTACA |
| ***TRIM44***  *NM_017583* | F’- CTGTGTCCTGTGTCCAGTCATTG  R’- GGCTTCGTCTAGGGTGGAGAGT |
| ***TRIM45***  *NM_025188* | F’- TGGGAACTCAGGCAAGACTCA  R’- CAGACACGTGGTGCAAACTGT |
| ***TRIM46***  *NM_025058* | F’- TGCCGAGGATGCCACAGT  R’- TTGCCCATGCCAATGGTT |
| ***TRIM47***  *NM_033452* | F’- GACAGCGACACAGCAGACAAG  R’- GTTGATAGGACACAGCACCCTCTT |
| ***TRIM48***  *NM_02411* | F’- TGCCCCATCTGCATGAACTA  R’- AGAAACAGGGCCTGCAAAAG |
| ***TRIM49***  *NM_020358* | F’- CACAAAGTCAACCGAGCAGATAA  R’- TCTGGCAAGAGAAGCCATCTTC |
| ***TRIM50***  *NM_178125* | F’- CCCGTCTCCACCGTCTACAG  R’- CCTTTTTCTGCTCCTGCTTCA |
| ***TRIM51***  *NM_032681* | F’- CAACGCGGCAGAGAAACC  R’- TGGCAATGAAAGCCATGTTC |
| ***TRIM52***  *NM_032765* | F’- GCTTTCGTCCCAACTTGCA  R’- GGGCACATCTGGCGAATTAT |
| ***TRIM53, TRIM53P***  *NM_001145126* | F’- CCTTGCCAGAAAAGCCAGTCT  R’- TGTGAGTGCCACACATTTGCT |
| ***TRIM54***  *NM_032546* | F’- TGCCATCCAGTCCATGGAA  R’- CCGACCTTATTGATCAGCTCCTT |
| ***TRIM55***  *NM_184085* | F’- CAACTCATCTGTCCCATCTGCTT  R’-TGTGCTGACAAGGGAGAATCAC |
| ***TRIM56***  *NM_030961* | F’- GCTCCGTGTCTGTGGATAAGAAG  R’- CCGTGGTAGGCTGTCAGGAA |
| ***TRIM58***  *NM_015431* | F’- AACCCTGAGCGATTTGACACA  R’- TGCCTCCCTGATGAGAAGCT |
| ***TRIM59***  *NM_173084* | F’- GAGACCTTTACGAATTCCACTCAAG  R’- TGCCAGTTGGAGCAATTTCA |
| ***TRIM60***  *NM_152620* | F’- TCGCTCCTGCCTCAGTGTATC  R’- TGAAGCTCTTGTATGGAAAGCAAA |
| ***TRIM61***  *NM_001012414* | F’- CCCATCTGTCTGGACTACTTGAAA  R’- GATGCAGGAGAGACAGAAGTTATGC |
| ***TRIM62***  *NM_018207* | F’- TTGTGGCTTACGGCAACTTG  R’- TCCACATCGAAGCGCTTTG |
| ***TRIM63***  *NM_032588* | F’- GACAGAGCAGGGCTTTGAGAAC  R’- TTCCTCATCTGTCCCAAAGTCA |
| ***TRIM64***  *NM_001136486* | F’- CAAGCATTACTGGGAGGTGGAT  R’- GGAATCTTGACAGACTCCCAGAA |
| ***TRIM65***  *NM_173547* | F’- TGGGTCTCCGGCAAGTTC  R’- CGTCTTGGCCACCTCGAT |
| ***TRIM66***  *NM_014818* | F’- CCCGGCATTATTACCAGATTATCA  R’- GGTATAGTGAGCTGGGTCCTTCTTT |
| ***TRIM67***  *NM_001004342* | F’- GGAAAACGGACTGGACTACGAA  R’- TGACGAGTTAAAGCATCCACAAG |
| ***TRIM68***  *NM_018073* | F’- GGAGGCAGCGGAGACCAT  R’- GGACCTGGCTCTGCTGGAT |
| ***TRIM69***  *NM_182985* | F’- AATCTCTGATGCTGTCCATTTCTTC  R’- TTGTCTCCAGTTGACCCTGTTG |
| ***TRIM71***  *NM_001039111* | F’- TGCGGCAAAACCTCAACA  R’-CTCGGGCCAGTAGGATGTCTAG |
| ***TRIM72***  *NM_001008274* | F’-CCGCCCGTCTGGACATC  R’-ATCAGAGCCCGGAACATCTTC |
| ***TRIM73***  *NM_198924* | F’- TGAAGCAGGAGCAGAAGAAGGT  R’- TCCGACTCATTGACGATTCG |

**Supplementary Table S2.** **Correlation of the relative expression of select TRIM genes with SLE^a^**

| **SLE Clinical presentation** | ***TRIM5*** | | ***TRIM11*** | | ***TRIM21*** | | ***TRIM72*** | |
| --- | --- | --- | --- | --- | --- | --- | --- | --- |
| **Variables** | **Spearman's rho** | **p-value** | **Spearman's rho** | **p-value** | **Spearman's rho** | **p-value** | **Spearman's rho** | **p-value** |
| **Fever** | -0.135 | 0.454 | -0.014 | 0.941 | -0.412 | **0.017** | -0.311 | 0.079 |
| **Acute skin: Malar rash** | -0.006 | 0.972 | -0.275 | 0.122 | -0.147 | 0.414 | 0.134 | 0.456 |
| **Photosensitive** | -0.059 | 0.743 | 0.045 | 0.806 | -0.022 | 0.902 | 0.089 | 0.622 |
| **Hair loss** | 0.265 | 0.137 | -0.179 | 0.319 | 0.086 | 0.636 | -0.039 | 0.830 |
| **Oral ulcer** | 0.182 | 0.312 | 0.107 | 0.552 | -0.008 | 0.964 | -0.083 | 0.648 |
| **Arthritis** | 0.102 | 0.571 | -0.173 | 0.337 | -0.077 | 0.671 | 0.051 | 0.777 |
| **Serositis** | -0.041 | 0.820 | 0.091 | 0.615 | -0.050 | 0.784 | -0.041 | 0.820 |
| **Neurological symptoms** | -0.133 | 0.459 | 0.067 | 0.712 | -0.307 | 0.082 | -0.093 | 0.605 |
| **Nephritis** | 0.071 | 0.695 | -0.142 | 0.430 | 0.107 | 0.555 | 0.080 | 0.659 |
| **Leukopenia Lymphopenia** | -0.358 | **0.041** | 0.135 | 0.454 | -0.135 | 0.454 | -0.209 | 0.242 |
| **Hemolytic anemia** | 0.019 | 0.915 | 0.206 | 0.250 | -0.039 | 0.831 | 0.328 | 0.062 |
| **Thrombocytopenia** | -0.262 | 0.141 | 0.300 | 0.089 | -0.198 | 0.269 | 0.089 | 0.620 |

^a^Spearman correlation; significance: *p* < 0.05.

**Supplementary Table S3. Correlations between *TRIM* expression and laboratory parameters in patients with SLE^a^**

| **SLE Clinical presentation** | ***TRIM5*** | | ***TRIM11*** | | ***TRIM21*** | | ***TRIM72*** | |
| --- | --- | --- | --- | --- | --- | --- | --- | --- |
| **Variables** | **r or rho** | **p-value** | **r or rho** | **p-value** | **r or rho** | **p-value** | **r or rho** | **p-value** |
| **C3** | -0.077 | 0.669 | 0.290 | 0.102 | 0.021 | 0.908 | 0.095 | 0.598 |
| **C4** | 0.084 | 0.642 | 0.160 | 0.375 | 0.069 | 0.705 | 0.104 | 0.564 |
| **DPL** | 0.177 | 0.325 | -0.149 | 0.409 | 0.150 | 0.406 | 0.089 | 0.624 |
| **Cr** | -0.067 | 0.710 | -0.148 | 0.411 | -0.065 | 0.720 | -0.128 | 0.477 |
| **GFR** | -0.082 | 0.651 | 0.114 | 0.529 | 0.001 | 0.996 | 0.007 | 0.968 |
| **Anti-dsDNA** | 0.307 | 0.082 | -0.133 | 0.460 | -0.087 | 0.630 | -0.086 | 0.632 |
| **Anti-SS-A^b^** | 0.210 | 0.240 | -0.006 | 0.972 | -0.032 | 0.860 | 0.083 | 0.647 |

^a^Pearson correlation (r); significance: *p* < 0.05.

^b^Spearman correlation (rho); significance: *p* < 0.05.

DPL, daily protein loss; Cr, creatinine; GFR, glomerular filtration rate.

**Supplementary Figure S1**


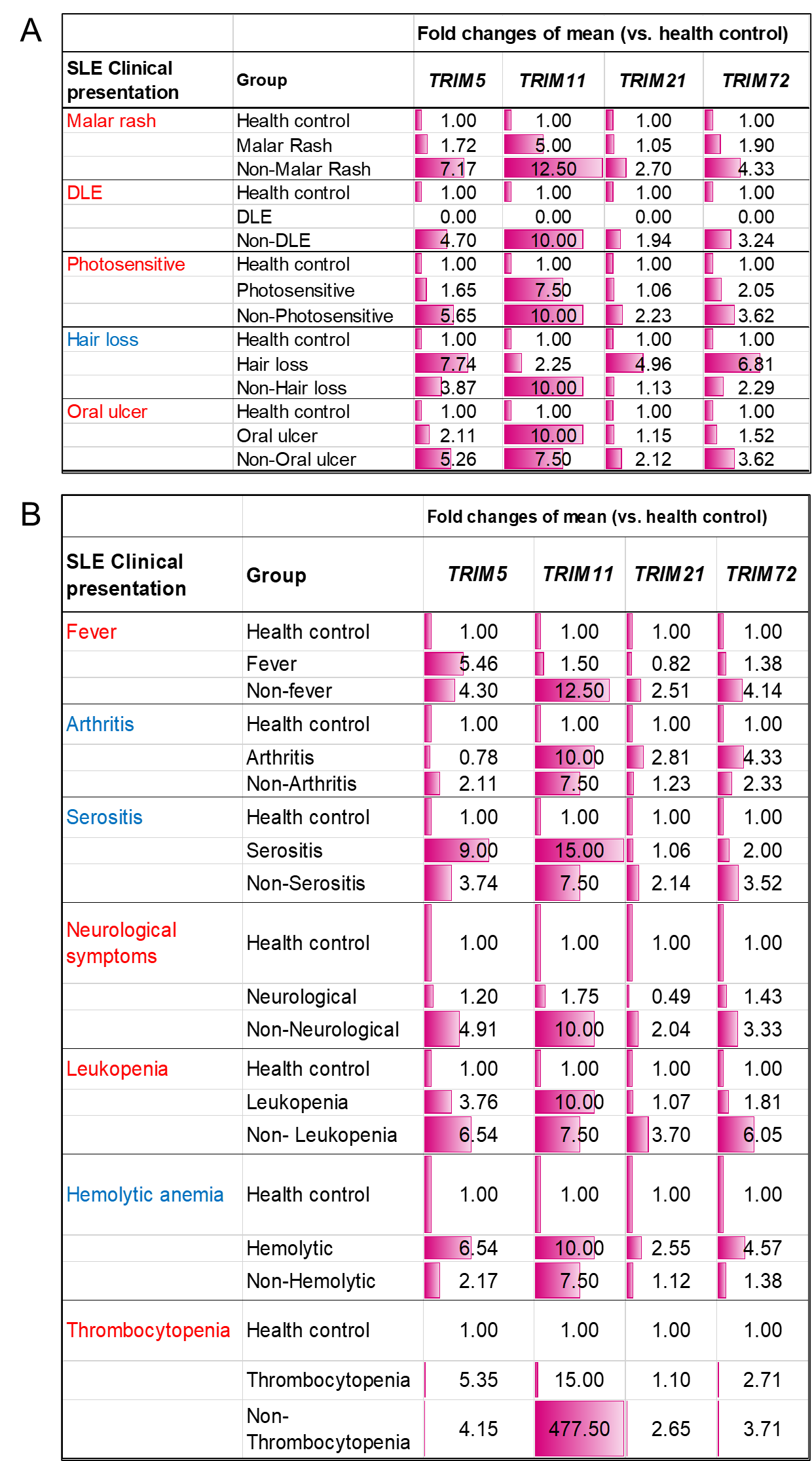


**Supplementary Fig. S1. Expression of TRIM genes across SLE clinical manifestations.**

**(A and B)** Fold changes in the mean expression levels of *TRIM5*, *TRIM11*, *TRIM21*, and *TRIM72* in patients with SLE stratified by clinical presentation relative to corresponding levels in healthy controls. Values represent the fold change in the mean expression level of each gene in the patient group relative to the corresponding level in the healthy control group, which is normalized to 1.00 across all categories, and are displayed as bar charts.
